# Homeostasis Model Assessment for Insulin Resistance Mediates the Positive Association of Triglycerides with Diabetes

**DOI:** 10.3390/diagnostics14070733

**Published:** 2024-03-29

**Authors:** Yutang Wang, Yan Fang, Michal Vrablik

**Affiliations:** 1 Discipline of Life Science, Institute of Innovation, Science and Sustainability, Federation University Australia, Ballarat, VIC 3350, Australia; 2 Third Department of Medicine, General University Hospital and First Faculty of Medicine, Charles University, 121 08 Prague, Czech Republic; michal.vrablik@vfn.cz

**Keywords:** insulin resistance, lipoprotein, diabetes, mediation analysis

## Abstract

Elevated circulating triglyceride levels have been linked to an increased risk of diabetes, although the precise mechanisms remain unclear. This study aimed to investigate whether low-density lipoprotein (LDL) cholesterol, homeostatic model assessment (HOMA) for insulin resistance, and C-reactive protein (CRP) served as mediators in this association across a sample of 18,435 US adults. Mediation analysis was conducted using the PROCESS Version 4.3 Macro for SPSS. Simple mediation analysis revealed that all three potential mediators played a role in mediating the association. However, in parallel mediation analysis, where all three mediators were simultaneously included, HOMA for insulin resistance remained a significant mediator (indirect effect coefficient, 0.47; 95% confidence interval [CI], 0.43–0.52; *p* < 0.05) after adjusting for all tested confounding factors. Conversely, LDL cholesterol (indirect effect coefficient, −0.13; 95% CI, −0.31–0.05; *p* > 0.05) and C-reactive protein (indirect effect coefficient, 0.01; 95% CI, −0.003–0.02; *p* > 0.05) ceased to be significant mediators. HOMA for insulin resistance accounted for 49% of the association between triglycerides and diabetes. In conclusion, HOMA for insulin resistance was the dominant mediator underlying the association between triglycerides and diabetes. Therefore, reducing triglyceride levels may hold promise for improving insulin sensitivity in diabetic patients.

## 1. Introduction

The prevalence of diabetes is on the rise, currently affecting 11.6% of the US population [1]. Diabetes stands as a significant contributor to blindness, kidney failure, heart attacks, stroke, and lower limb amputation [2], ranking as the eighth leading cause of death in the US, with approximately 103,000 deaths per year attributed to it [1]. The economic burden of diabetes care weighs heavily on society, with direct medical costs estimated at USD 306.6 billion per year in the US alone [1]. Thus, understanding the pathogenesis of the disease and developing new preventative and therapeutic strategies are imperative.

A plethora of studies suggests that higher triglyceride levels may promote the development and progression of diabetes. Elevated triglycerides correlate with increased diabetes prevalence [3,4,5], incidence [6,7], and mortality [8]. Notably, a genetic study revealed that alleles associated with higher triglycerides heightened the risk of diabetes [9]. Moreover, fenofibrate, a triglyceride-lowering medication, demonstrated glucose-lowering effects in diabetic mice [10], hinting at a potential causal link between higher triglycerides and diabetes.

However, the precise mechanism bridging triglycerides and diabetes remains elusive. Low-density lipoprotein (LDL) cholesterol [11], insulin resistance [12,13], and inflammation [14,15] emerge as potential players in elucidating this association.

The association between high triglycerides and elevated LDL cholesterol in elderly individuals hints at a possible involvement of LDL cholesterol [16]. Mechanistically, triglyceride-rich very-low-density lipoprotein (VLDL) can form LDL through delipidation [17]. Epidemiological evidence suggests a link between high triglycerides and insulin resistance, as evidenced by positive associations with homeostatic model assessment (HOMA) for insulin resistance in diverse populations worldwide, including the US [8], South Korea [18], and China [19,20]. Likewise, inflammation might contribute to the triglyceride–diabetes nexus. High triglycerides can increase inflammation [11,21] and pose a risk for pancreatitis [22]. Furthermore, triglycerides correlate positively with C-reactive protein [23,24], a well-established inflammatory marker [25].

Nevertheless, questions linger: do LDL cholesterol, insulin resistance, and inflammation indeed mediate the association between triglycerides and diabetes? If so, to what extent do they mediate this link? The current study aimed to address these questions by examining a large group of US adults who participated in the National Health and Nutrition Examination Survey (NHANES) from 1988 to 2014.

## 2. Materials and Methods

### 2.1. Study Participants

**Figure 1:**
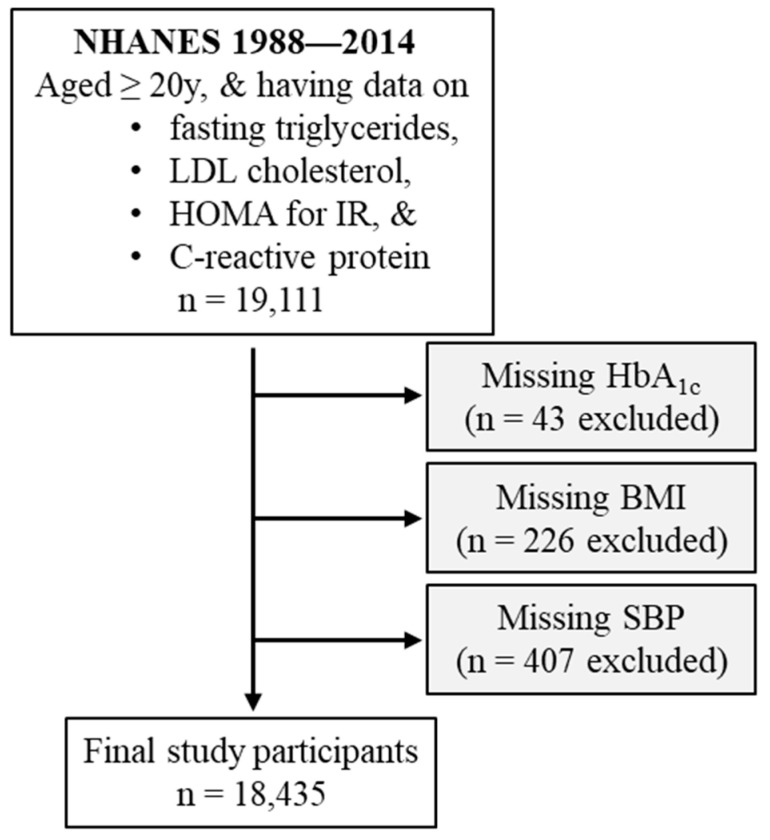
Flow diagram of the study participants. BMI, body mass index; HbA_1c_, hemoglobin A_1c_; HOMA for IR, homeostatic model assessment for insulin resistance; LDL, low-density lipoprotein; NHANES, National Health and Nutrition Examination Survey; SBP, systolic blood pressure.

This study included participants from NHANES III (1988–1994) and the subsequent eight cycles of NHANES from 1999 to 2014. NHANES was designed to assess the health and nutritional status of the civilian noninstitutionalized US population. It employed a complex, multistage probability sampling design to select a participant sample that was representative of the population. About 83% of the initially invited individuals participated in the data collection. The survey was well planned by the National Center for Health Statistics (NCHS) within the Centers for Disease Control and Prevention (CDC) [26]. The inclusion criteria included age of ≥20 years and the presence of the following data: fasting triglycerides, LDL cholesterol, HOMA for insulin resistance, and C-reactive protein. This resulted in a group of 19,111 participants. The following participants were excluded from the analysis: those without blood hemoglobin A_1c_ (HbA_1c_, *n* = 43), body mass index (*n* = 226), or systolic blood pressure (*n* = 407). Therefore, the remaining 18,435 participants were included in the final analysis (Figure 1).

### 2.2. Exposure Variable

The exposure variable of this study was triglyceride levels in fasting serum. Fasting blood was collected from participants with a time between 8.0 and 23.9 h after their last caloric intake [8,27,28]. The concentrations of triglycerides in the serum were measured using an enzymatic method which employed a series of coupled reactions in which triglycerides were hydrolyzed to produce glycerol [29]. The resultant glycerol was then phosphorylated and oxidized to produce hydrogen peroxide. The formed hydrogen peroxide was then converted by peroxidase to form a color product that was measured using a spectrophotometer at a wavelength of 500 nm.

### 2.3. Outcome Variable

The outcome of the current study was diabetes, defined by a range of criteria, including a fasting plasma glucose level at or above 126 mg/dL, an HbA_1c_ level in whole blood at or above 6.5%, and a 2 h oral glucose tolerance test result at or above 200 mg/dL. In addition, the use of hypoglycemic medications and self-reported diagnosis of diabetes were also regarded as criteria for diabetes [30].

### 2.4. Candidate Mediators

#### 2.4.1. LDL Cholesterol

LDL cholesterol was retrieved directly from the NHANES website [31]. It was calculated according to the Friedewald formula based on total cholesterol, high-density lipoprotein (HDL) cholesterol, and triglyceride concentrations for those with triglycerides ≤ 400 mg/dL [32].

#### 2.4.2. HOMA for Insulin Resistance

HOMA for insulin resistance was calculated using the following published formula [33]: (serum insulin in µU/mL × plasma glucose in mmol/L)/22.5. The values of fasting plasma glucose and serum insulin were directly obtained from the NHANES website, and they were measured using the following methods.

The levels of glucose in the plasma were measured using the hexokinase-mediated reaction method, as described previously [34]. Briefly, hexokinase catalyzed glucose to produce glucose-6-phosphate. In the presence of nicotinamide adenine dinucleotide (NAD), the enzyme glucose-6-phosphate dehydrogenase catalyzed glucose-6-phosphate to generate 6-phosphogluconate, in which process, NAD was reduced to the reduced form of nicotinamide adenine dinucleotide (NADH). The resultant increases in NADH levels were proportional to the plasma glucose concentrations and were measured using a spectrophotometer at a wavelength of 340 nm [35].

Insulin levels in the serum were measured by an immunoenzymometric assay [36]. Briefly, insulin was captured by binding with a non-labeled monoclonal antibody immobilized on a magnetic solid phase, and the captured insulin was then bound with another enzyme-labeled monoclonal antibody. Magnetic beads containing insulin and bound enzyme-labeled monoclonal antibodies were incubated with 4-methylumbelliferyl phosphate, a fluorogenic substrate. The fluorescence intensity produced at a certain reaction time was proportional to the insulin concentration in the serum.

#### 2.4.3. C-Reactive Protein

C-reactive protein in the serum was measured using the latex-enhanced nephelometry method [37]. Briefly, a dilute solution of the serum sample was mixed with latex particles, and the latter were coated with monoclonal anti-C-reactive protein antibodies that were generated from mice. C-reactive protein present in the test sample formed an antigen–antibody complex with the latex particles. The resultant complex would scatter light. The extent of light scattering was then measured using a nephelometric procedure after 6 min, and it was proportional to the C-reactive protein concentrations in the testing samples.

### 2.5. Confounding Covariables

Details of confounding covariables were described in previous publications [5,38]. The list included age, sex, ethnicity, body mass index, education, poverty–income ratio, survey periods, physical activity, alcohol consumption, smoking, systolic blood pressure, total cholesterol, HDL cholesterol, and family history of diabetes. Among these variables, age, body mass index, systolic blood pressure, total cholesterol, and HDL cholesterol were continuous variables.

### 2.6. Statistical Analyses

The presentation of data involved reporting numbers (with percentages) for categorical variables, medians (with interquartile ranges) for continuous variables that were not normally distributed, and means (with standard deviations) for normally distributed continuous variables, to outline the characteristics of the participants [39]. Differences among categorical variables were assessed using Pearson’s chi-square analysis [40], while differences in continuous variables were examined using one-way ANOVA tests for normally distributed variables and Kruskal–Wallis one-way ANOVA tests for those not following a normal distribution [41].

**Figure 2:**
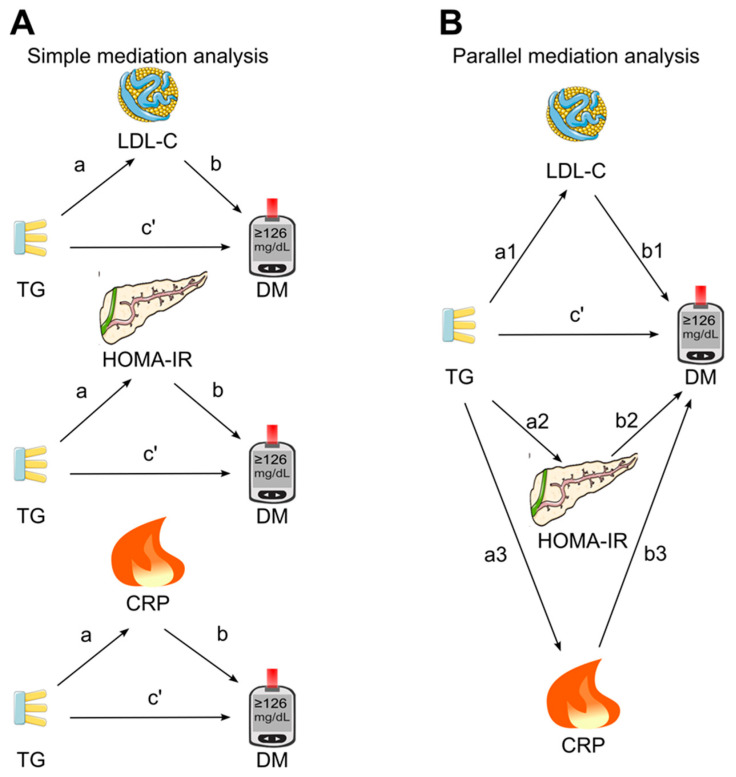
Mediation analysis models. (**A**) Simple mediation. LDL cholesterol, HOMA for insulin resistance, or C-reactive protein was added as single mediator for the association of triglycerides with diabetes. (**B**) Parallel mediation. In this analysis, LDL cholesterol, HOMA for insulin resistance, and C-reactive protein were added simultaneously to assess their mediation effects on the association of triglycerides with diabetes. a, association coefficient between triglycerides and the tested mediator; b, association coefficient between the tested mediator and diabetes; c’, also known as direct effect, referring to the association coefficient between triglycerides and diabetes in the presence of the tested mediator (simple mediation) or all tested mediators (parallel mediation); CRP, C-reactive protein; DM, diabetes; HOMA-IR, homeostasis model assessment for insulin resistance; LDL-C, low-density lipoprotein cholesterol; TG, triglycerides. This figure was partly generated using Servier Medical Art, provided by Servier, licensed under a Creative Commons Attribution 3.0 unported license.

The association between triglycerides and diabetes was analyzed using binary logistic regression [42], with or without adjustment for confounding factors. Mediation analysis was conducted using the PROCESS Version 4.3 Macro for SPSS [43], a regression path analysis modeling tool that was accessible via the processmacro website [44]. At the first stage, a simple mediation analysis was conducted (Figure 2A), in which the three candidate mediators (LDL cholesterol, HOMA for insulin resistance, and C-reactive protein) were put into the model separately to investigate the individual mediation effects on the association between triglycerides and diabetes. Secondly, parallel mediation analysis was employed (Figure 2B), in which all three candidate mediators were simultaneously put into the model.

The association coefficients a (between the triglycerides and the tested mediator) and b (between the tested mediator and diabetes) were generated by mediation analysis (Figure 2). The direct effect (c’) was the association coefficient between triglycerides and diabetes in the presence of the tested mediator(s). The indirect effect, or mediation effect, was calculated by multiplying a and b (a × b). The 95% confidence interval (CI) was generated using the bootstrapping method [45] with 5000 samples to assess the significance of the mediating effects [46]. The mediation effect (a × b) was regarded as significant (*p* < 0.05) if the 95% CI did not encompass zero [47]. The proportion mediated (PM) was calculated using the formula a × b/(a × b + c’) and it provided an estimate of the extent to which the association between triglycerides and diabetes was accounted for by the pathway through the tested mediator [48].

The not normally distributed variables underwent natural log-transformation to enhance data distribution prior to inclusion in regression and mediation analysis models [49]; these variables included triglycerides, HOMA for insulin resistance, C-reactive protein, body mass index, systolic blood pressure, total cholesterol, and HDL cholesterol. The null hypothesis was rejected for two-tailed *p*-values < 0.05. Statistical analyses were conducted using SPSS version 27.0 (IBM SPSS Statistics for Windows, Armonk, NY, USA, IBM Corporation).

## 3. Results

### 3.1. General Characteristics

This study included 18,435 US adult participants including 2550 individuals with diabetes. The participants had a mean (standard deviation) age of 49 (19) years. Individuals with higher fasting triglycerides had a higher prevalence of diabetes. Higher triglycerides were accompanied by higher levels of LDL cholesterol, HOMA for insulin resistance, C-reactive protein, body mass index, systolic blood pressure, and total cholesterol, as well as lower levels of HDL cholesterol. Those with higher triglycerides were older and had less education and income (Table 1).

### 3.2. Association of Triglycerides with Diabetes Diagnosis

A 1-natural-log-unit increase in triglycerides was associated with a 2.54-fold higher risk of diabetes (odds ratio, OR, 2.54; 95% CI, 2.23–2.89; *p* < 0.001; Model 5, Table 2) after adjustment for risk factors except for the tested mediators (i.e., LDL cholesterol, HOMA for insulin resistance, and C-reactive protein). After further adjustment for these three tested mediators, a 1-natural-log-unit increase in triglycerides remained associated with a higher risk of diabetes (OR, 1.88; 95% CI, 1.48–2.37; *p* < 0.001; Model 9, Table 2). This suggested that if LDL cholesterol, HOMA for insulin resistance, or C-reactive protein mediated the association between triglycerides and diabetes in subsequent analyses, the mediation would be partial rather than complete [50].

### 3.3. Mediation Analyses of the Association of Triglycerides with Diabetes

**Figure 3:**
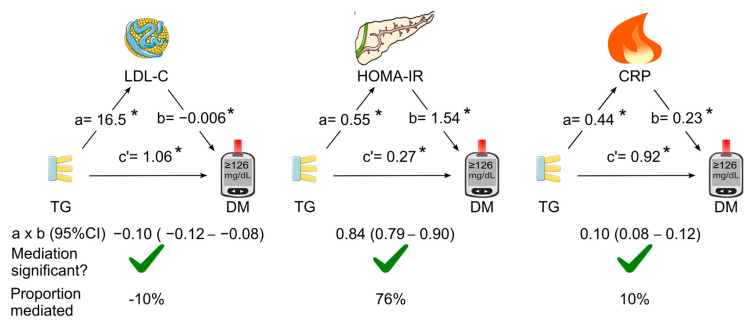
Simple mediation analysis. LDL cholesterol, HOMA for insulin resistance, or C-reactive protein was added as single mediator for the association of triglycerides with diabetes. a, association coefficient between triglycerides and the tested mediator; b, association coefficient between the tested mediator and diabetes; c’, association coefficient between triglycerides and diabetes in the presence of the tested mediator; CI, confidence interval; CRP, C-reactive protein; DM, diabetes; HOMA-IR, homeostasis model assessment for insulin resistance; LDL-C, low-density lipoprotein cholesterol; TG, triglycerides. Green tick = yes. * *p* < 0.05. This figure was partly generated using Servier Medical Art, provided by Servier, licensed under a Creative Commons Attribution 3.0 unported license.

The mediation coefficients of LDL cholesterol, HOMA for insulin resistance, and C-reactive protein for the association between triglycerides and diabetes are listed in Table 1. When LDL cholesterol, HOMA for insulin resistance, and C-reactive protein were added as single mediators in the mediation analysis (simple mediation), all of the three tested mediators were found to play a role in mediating the association between triglycerides and diabetes (Figure 3). HOMA for insulin resistance was the dominant mediator (indirect effect coefficient, 0.84; 95% CI, 0.79–0.90; *p* < 0.05), which accounted for 76% of the association. However, LDL cholesterol negatively mediated the association by 10% (Figure 3).

**Figure 4:**
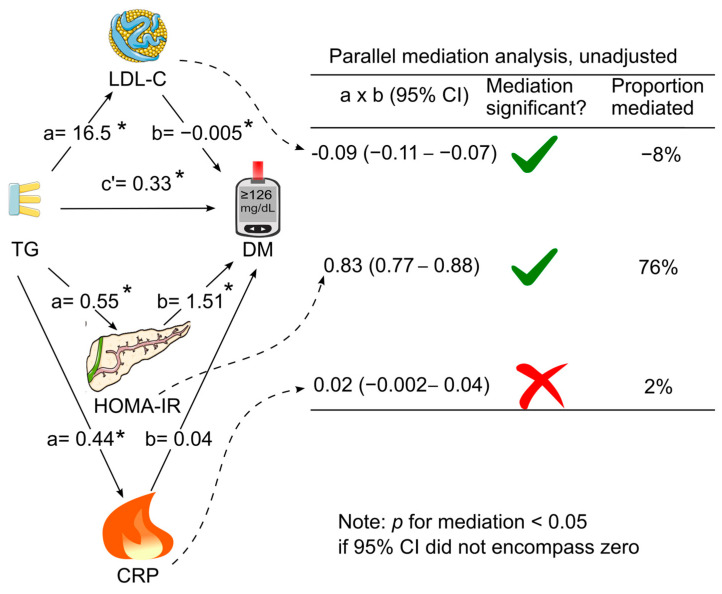
Unadjusted parallel mediation analysis. LDL cholesterol, HOMA for insulin resistance, and C-reactive protein were added as parallel mediators for the association of triglycerides with diabetes without adjustment for confounding factors. a, association coefficient between triglycerides and the tested mediator; b, association coefficient between the tested mediator and diabetes; c’, association coefficient between triglycerides and diabetes in the presence of all the tested mediators; CI, confidence interval; CRP, C-reactive protein; DM, diabetes; HOMA-IR, homeostasis model assessment for insulin resistance; LDL-C, low-density lipoprotein cholesterol; TG, triglycerides. Green tick = yes. Red cross = no. * *p* < 0.05. This figure was partly generated using Servier Medical Art, provided by Servier, licensed under a Creative Commons Attribution 3.0 unported license.

**Figure 5:**
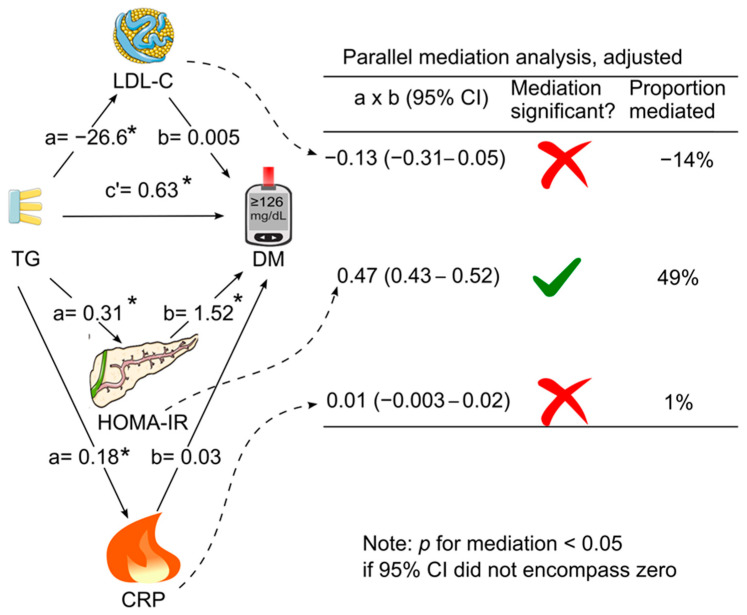
Parallel mediation analysis with adjustment for confounding factors. LDL cholesterol, HOMA for insulin resistance, and C-reactive protein were placed simultaneously into the analysis as parallel mediators for the association of triglycerides with diabetes. This analysis was adjusted for confounding factors. The latter confounding factors included age, sex, ethnicity, body mass index, poverty–income ratio, education, survey period, lifestyle confounding factors (physical activity, alcohol consumption, and smoking status), clinical confounding factors (systolic blood pressure, total cholesterol, HDL cholesterol, and family history of diabetes), and fasting time. Abbreviations: a, association coefficient between triglycerides and the tested mediator; b, association coefficient between the tested mediator and diabetes; c’, association coefficient between triglycerides and diabetes in the presence of the tested mediators; CI, confidence interval; CRP, C-reactive protein; DM, diabetes; HOMA-IR, homeostasis model assessment for insulin resistance; LDL-C, low-density lipoprotein cholesterol; TG, triglycerides. The green tick represents yes and the red cross represents no. * *p* < 0.05. This figure was partly produced using Servier Medical Art which was licensed under a Creative Commons Attribution 3.0 unported license.

When LDL cholesterol, HOMA for insulin resistance, and C-reactive protein were added simultaneously as mediators in the same model (parallel mediation analysis), in the absence of adjustment for confounding factors, only HOMA for insulin resistance and LDL cholesterol mediated the association between triglycerides and diabetes (Figure 4). HOMA for insulin resistance remained the dominant mediator, which accounted for 76% of the association, whereas LDL cholesterol negatively mediated the association by 8% (Figure 4). After further adjustment for all the tested confounding factors, only HOMA for insulin resistance mediated the association between triglycerides and diabetes (indirect effect coefficient, 0.47; 95% CI, 0.43–0.52; *p* < 0.05), accounting for 49% of the association (Table 3 & Figure 5).

## 4. Discussion

Utilizing a robust sample of US adults (*n* = 18,435), this study revealed that HOMA for insulin resistance partially mediated the association between fasting triglycerides and diabetes, explaining 49% of the association after adjusting for confounding factors. Notably, LDL cholesterol and C-reactive protein did not exhibit significant mediation effects.

It has been well known that triglycerides are positively associated with insulin resistance in humans [8,18,19,20], which is confirmed by the current study. Consistently, pharmacological interventions, such as fenofibrate alone or in combination with omega-3 fatty acids, improve insulin sensitivity in individuals with hypertriglyceridemia [51]. However, the precise mechanism linking triglycerides to insulin resistance remains elusive. Proposed mechanisms include impediments to glucose transport [52], hindrance of glucose oxidation [53], and decreased glycogen synthesis [54], collectively resulting in diminished cellular response to insulin.

Insulin resistance mediated 49% of the association between triglycerides and diabetes. Approximately 55% of type-2 diabetes patients from 11 European countries exhibit elevated triglyceride levels (>150 mg/dL) [55]. The current study showed that 46% of US patients with diabetes had elevated triglyceride levels. Thus, reducing triglycerides could hold therapeutic promise in enhancing insulin sensitivity, an avenue yet to be recognized by the American Diabetes Association [56]. Further research is needed to establish the role of lowering triglycerides in glycemic control.

High triglycerides have been implicated in inflammation [11,21], as evidenced by positive associations with C-reactive protein [23,24] and pancreatitis risk [57,58]. In addition, pharmacologically lowering triglycerides decreases circulating levels of inflammatory markers including C-reactive protein and fibrinogen [51]. Yet, C-reactive protein failed to contribute significantly to the association between triglycerides and diabetes in this study, suggesting that inflammation might not play a significant role in high-triglyceride-induced diabetes. Higher triglycerides have been shown to be positively associated with other inflammatory markers including interleukin 6 (IL-6) [59] and fibrinogen [25,51]. Therefore, exploration into the mediating roles of other inflammatory markers like interleukin 6 (IL-6) and fibrinogen remains warranted.

Elevated triglycerides often coexist with high LDL cholesterol in elderly individuals, possibly due to the conversion of VLDL to LDL [17]. Interestingly, after adjustment, LDL cholesterol marginally diminished the association between triglycerides and diabetes by 14%, albeit not reaching statistical significance. The competing effect of LDL cholesterol against triglycerides has been reported previously. For example, a 1-natural-log increase in triglycerides was significantly associated with an increased risk of coronary heart disease mortality (relative risk, 1.86; 95% CI, 1.12–3.08) in male participants with lower LDL cholesterol (<160 mg/dL) [60]; however, the associated risk was no longer significant (relative risk, 1.13; 95% CI, 0.64–1.98) in those with higher LDL cholesterol (≥160 mg/dL).

The underlying reasons for LDL cholesterol’s slight competition with triglycerides in diabetes risk remain unclear. It is plausible that LDL cholesterol exerts an opposing effect on diabetes compared to triglycerides. Indeed, statin therapy, which lowers LDL cholesterol, has been associated with a 26% increase in the risk of new-onset diabetes [61,62], while fenofibrate, a triglyceride-lowering agent, demonstrated glucose-lowering effects in mice with type-2 diabetes [10].

Strengths of this study include its sizable sample and adjustment for various confounding factors, including body mass index, systolic blood pressure, total cholesterol, and HDL cholesterol. However, its reliance on US participants may limit generalizability to other populations.

## 5. Conclusions

This study hypothesized that LDL cholesterol, insulin resistance, and C-reactive protein mediated the association between triglycerides and diabetes. The results showed that HOMA for insulin resistance, but not LDL cholesterol or C-reactive protein, is the primary mediator linking triglycerides and diabetes. Consequently, reducing triglyceride levels could play a pivotal role in enhancing insulin sensitivity among diabetic patients, thereby contributing to glycemic control.

## Figures and Tables

**Table 1:** Characteristics of the 18,435 participants, stratified according to observed quartiles of triglycerides.

	Quartile 1	Quartile 2	Quartile 3	Quartile 4	Overall	*p* Value
Sample size	4738	4569	4523	4605	18,435	NA
Diabetes, *n* (%)	320 (6.8)	519 (11.4)	698 (15.4)	1013 (22.0)	2550 (13.8)	<0.001
Triglycerides, mg/dL, median (IQR)	63 (53–72)	95 (87–104)	134 (123–147)	210 (183–255)	112 (79–162)	<0.001
LDL cholesterol, mg/dL, mean (SD)	107.5 (32.2)	121.4 (34.2)	129.3 (36.1)	129.5 (40.5)	121.8 (37.0)	<0.001
HOMA-IR, median (IQR)	1.6 (1.1–2.5)	2.1 (1.4–3.2)	2.6 (1.7–4.1)	3.3 (2.2–5.4)	2.3 (1.5–3.7)	<0.001
CRP, mg/dL, median (IQR)	0.21 (0.09–0.30)	0.21 (0.14–0.42)	0.21 (0.18–0.55)	0.26 (0.21–0.60)	0.21 (0.15–0.47)	<0.001
Glucose, mg/dL, median (IQR)	93 (88–100)	96 (90–104)	99 (92–108)	101 (93–113)	97 (90–106)	<0.001
Insulin, uU/mL, median (IQR)	7.1 (5.1–10.2)	8.5 (6.0–12.8)	10.4 (7.2–15.5)	12.7 (8.8–19.1)	9.4 (6.4–14.4)	<0.001
HbA_1c_, %, median (IQR)	5.3 (5.0–5.6)	5.4 (5.1–5.7)	5.4 (5.1–5.8)	5.5 (5.2–5.9)	5.4 (5.1–5.7)	<0.001
BMI, kg/m^2^, median (IQR)	24.9 (21.9–28.8)	26.4 (23.3–30.3)	27.9 (24.6–31.8)	29.0 (26.0–32.9)	27.1 (23.8–31.2)	<0.001
SBP, mm Hg, median (IQR)	115 (107–127)	120 (110–133)	123 (113–137)	125 (114–139)	121 (111–134)	<0.001
HDL cholesterol, mg/dL, median (IQR)	58 (49–70)	53 (44–63)	48 (41–58)	43 (36–52)	50 (42–62)	<0.001
Total cholesterol, mg/dL, median (IQR)	178 (156–202)	194 (170–219)	204 (180–232)	217 (191–246)	197 (171–226)	<0.001
Age, y, mean (SD)	43 (18)	49 (19)	52 (18)	52 (18)	49 (19)	<0.001
Sex (male), *n* (%)	2004 (42.3)	2174 (47.6)	2194 (48.5)	2333 (50.7)	8705 (47.2)	<0.001
Ethnicity, *n* (%)						
Non-Hispanic white	1930 (40.7)	2161 (47.3)	2251 (49.8)	2362 (51.3)	8704 (47.2)	<0.001
Non-Hispanic black	1637 (34.6)	1055 (23.1)	725 (16.0)	487 (10.6)	3904 (21.2)	
Hispanic	1030 (21.7)	1213 (26.5)	1400 (31.0)	1613 (35.0)	5256 (28.5)	
Other	141 (3.0)	140 (3.1)	147 (3.3)	143 (3.1)	571 (3.1)	
Education, *n* (%)						
<High school	1269 (26.8)	1460 (30.2)	1564 (34.6)	1805 (39.2)	6098 (33.1)	<0.001
High school	1224 (25.8)	1190 (26.0)	1201 (26.6)	1207 (26.2)	4822 (26.2)	
>High school	2236 (47.2)	1906 (41.7)	1743 (38.5)	1585 (34.4)	7470 (40.5)	
Unknown	9 (0.2)	13 (0.3)	15 (0.3)	8 (0.2)	45 (0.2)	
Poverty–income ratio, *n* (%)						
<130%	1226 (25.9)	1175 (25.7)	1188 (26.3)	1350 (29.3)	4939 (26.S8)	<0.001
130–349%	1752 (37.0)	1722 (37.7)	1781 (39.4)	1718 (37.3)	6973 (37.8)	
≥350%	1358 (28.7)	1256 (27.5)	1200 (26.5)	1131 (24.6)	4945 (26.8)	
Unknown	402 (8.5)	416 (9.1)	354 (7.8)	406 (8.8)	1578 (8.6)	
Physical activity, *n* (%)						
Active	1385 (29.2)	1234 (27.0)	1056 (23.3)	1033 (22.4)	4708 (25.5)	<0.001
Insufficiently active	1921 (40.5)	1801 (39.4)	1863 (41.2)	1802 (39.1)	7387 (40.1)	
Inactive	1431 (30.2)	1531 (33.5)	1602 (35.4)	1767 (38.4)	6331 (34.3)	
Unknown	1 (0)	3 (0.1)	2 (0)	3 (0.1)	9 (0)	
Alcohol consumption, *n* (%)						
0 drink/week	762 (16.1)	838 (18.3)	880 (19.5)	931 (20.2)	3411 (18.5)	<0.001
<1 drink/week	1060 (22.4)	1071 (23.4)	1038 (22.9)	1012 (22.0)	4181 (22.7)	
1–6 drinks/week	1115 (23.5)	958 (21.0)	879 (19.4)	806 (17.5)	3758 (20.4)	
≥7 drinks/week	611 (12.9)	630 (13.8)	572 (12.6)	588 (12.8)	2401 (13.0)	
Unknown	1190 (25.1)	1072 (23.5)	1154 (25.5)	1268 (27.5)	4684 (25.4)	
Smoking status, *n* (%)						
Past smoker	963 (20.3)	1111 (24.3)	1009 (22.3)	1048 (22.8)	4131 (22.4)	<0.001
Current smoker	963 (20.3)	1114 (24.4)	1259 (27.8)	1414 (30.7)	4750 (25.8)	
Nonsmoker	2808 (59.3)	2343 (51.3)	2252 (49.8)	2140 (46.5)	9543 (51.8)	
Unknown	4 (0.1)	1 (0)	3 (0.1)	3 (0.1)	11 (0.1)	
Family history of diabetes, *n* (%)						
Yes	2012 (42.5)	1926 (42.2)	1973 (43.6)	2229 (48.4)	8140 (44.2)	<0.001
No	2615 (55.2)	2561 (56.1)	2471 (54.6)	2298 (49.9)	9945 (53.9)	
Unknown	111 (2.3)	82 (1.8)	79 (1.7)	78 (1.7)	350 (1.9)	

Abbreviations: BMI, body mass index; CRP, C-reactive protein; HbA_1c_, hemoglobin A_1c_; HDL, high-density lipoprotein; HOMA-IR, homeostatic model assessment for insulin resistance; IQR, interquartile range; LDL, low-density lipoprotein; *n*, number; NA, not applicable; SBP, systolic blood pressure; SD, standard deviation.

**Table 2:** Natural log-transformed triglycerides and risk for diabetes in 18,435 participants.

Models	Odds Ratio	95% CI	*p* Value
Model 1	2.69	2.47–2.93	<0.001
Model 2	2.60	2.36–2.86	<0.001
Model 3	2.18	1.97–2.42	<0.001
Model 4	2.58	2.28–2.93	<0.001
Model 5	2.54	2.23–2.89	<0.001
Model 6 (Model 5 + LDL-C)	2.95	2.37–3.68	<0.001
Model 7 (Model 5 + HOMA-IR)	1.61	1.40–1.85	<0.001
Model 8 (Model 5 + CRP)	2.55	2.24–2.90	<0.001
Model 9 (Model 5 + LDL-C + HOMA-IR + CRP)	1.88	1.48–2.37	<0.001

CI, confidence interval; CRP, C-reactive protein; HOMA-IR, homeostatic model assessment for insulin resistance; LDL-C, low-density lipoprotein cholesterol. Model 1: not adjusted; Model 2: adjusted for age, sex, and ethnicity; Model 3: adjusted for factors in Model 2 plus body mass index, poverty–income ratio, education, physical activity, alcohol consumption, smoking status, and survey period; Model 4: adjusted for factors in Model 3 plus systolic blood pressure, total cholesterol, and HDL cholesterol; Model 5: adjusted for factors in Model 4 plus family history of diabetes.

**Table 3:** Association coefficients of LDL cholesterol, HOMA for insulin resistance, and C-reactive protein for mediating the association between triglycerides and diabetes.

Tested Mediators	a (95% CI)	b (95% CI)	Direct Effect, c’ (95% CI)	Indirect (Mediation) Effect a × b (95% CI)
Unadjusted simple mediation ^1^				
LDL-C	16.5 (15.5–17.5) *	−0.006 (−0.007–−0.005) *	1.06 (0.98–1.15) *	−0.10 (−0.12–−0.08) *
HOMA-IR	0.55 (0.53–0.57) *	1.54 (1.46–1.61) *	0.27 (0.17–0.37) *	0.84 (0.79–0.90) *
CRP	0.44 (0.41–0.47) *	0.23 (0.19–0.27) *	0.92 (0.83–1.00) *	0.10 (0.08–0.12) *
Unadjusted parallel mediation ^2^				
LDL-C	16.5 (15.5–17.5) *	−0.005 (−0.007–−0.004) *	0.33 (0.23–0.43) *	−0.09 (−0.11–−0.07) *
HOMA-IR	0.55 (0.53–0.57) *	1.51 (1.43–1.59) *		0.83 (0.77– 0.88) *
CRP	0.44 (0.41–0.47) *	0.04 (−0.001–0.09)		0.02 (−0.002–0.04)
Adjusted parallel mediation ^3^				
LDL-C	−26.6 (−26.9–−26.3) *	0.005 (−0.001–0.011)	0.63 (0.40–0.86) *	−0.13 (−0.31–0.05)
HOMA-IR	0.31 (0.29–0.33) *	1.52 (1.42–1.61) *		0.47 (0.43–0.52) *
CRP	0.18 (0.14–0.22) *	0.03 (−0.02–0.09)		0.01 (−0.003–0.02)
Adjusted parallel mediation, with further adjustment for fasting time ^4^				
LDL-C	−26.6 (−26.9–−26.3) *	0.005 (−0.001–0.011)	0.63 (0.39–0.86) *	−0.13 (−0.31–0.05)
HOMA-IR	0.31 (0.29–0.33) *	1.52 (1.42–1.61) *		0.47 (0.43–0.52) *
CRP	0.18 (0.14–0.22) *	0.03 (−0.02–0.09)		0.01 (−0.003–0.02)

Abbreviations: a, association coefficient between triglycerides and the tested mediator; b, association coefficient between the tested mediator and diabetes; c’, also known as direct effect, referring to the association coefficient between triglycerides and diabetes in the presence of the tested mediator (simple mediation) or all the tested mediators (parallel mediation); CI, confidence interval; CRP, C-reactive protein; HOMA-IR, homeostasis model assessment for insulin resistance; LDL-C, low-density lipoprotein cholesterol. ^1^ LDL cholesterol, HOMA for insulin resistance, or C-reactive protein was added as single mediator in the mediation analysis. The analysis was unadjusted. ^2^ LDL cholesterol, HOMA for insulin resistance, and C-reactive protein were added simultaneously as parallel mediators in the mediation analysis. The analysis was unadjusted. ^3^ LDL cholesterol, HOMA for insulin resistance, and C-reactive protein were added simultaneously as parallel mediators in the mediation analysis. The analysis was adjusted for age, sex, ethnicity, body mass index, poverty–income ratio, education, physical activity, alcohol consumption, smoking status, survey period, systolic blood pressure, total cholesterol, HDL cholesterol, and family history of diabetes. ^4^ LDL cholesterol, HOMA for insulin resistance, and C-reactive protein were added simultaneously as parallel mediators in the mediation analysis. The analysis was adjusted for all the confounding factors in footnote 3 plus fasting time. * *p* < 0.05.

## Data Availability

All data in the current analysis are publicly available on the NHANES website (https://www.cdc.gov/nchs/nhanes/index.htm), accessed on 1 July 2022.
